# The adsorption characteristics of Cu(II) and Zn(II) on the sediments at the mouth of a typical urban polluted river in Dianchi Lake: taking Xinhe as an example

**DOI:** 10.1038/s41598-021-96638-4

**Published:** 2021-08-23

**Authors:** Xiang-shu Ma, Leng Liu, Yi-chuan Fang, Xiao-long Sun

**Affiliations:** 1 Yunnan Key Laboratory of Plateau Wetland Conservation, Restoration and Ecological Services, College of Wetlands, Southwest Forestry University, Kunming, 650224 China; 2 National Plateau Wetlands Research Center, Southwest Forestry University, Kunming, 650224 China; 3 National Wetland Ecosystem Fixed Research Station of Yunnan Dianchi, Southwest Forestry University, Kunming, 650224 China

**Keywords:** Environmental sciences, Environmental impact

## Abstract

This study is to determine the spatial distribution characteristics of Cu and Zn adsorption on the sediments of the estuary of Dianchi Lake, as well as the composite adsorption law of Cu and Zn on combinations of sediment organic matter, metal oxides, and organic–inorganic composites. The relationship between the adsorption contribution of each component of the substance. A static adsorption experiment was applied to the sediments in the estuary of Dianchi Lake. The relationship between adsorption capacity and sediment composition was analyzed through correlation analysis and redundant analysis. The results show that along the direction of the river flow and the vertical depth, the adsorption capacity presents a relatively obvious spatial distribution law; the change trend of sediment component content is not the same as the change trend of Cu and Zn adsorption capacity. The change trend of the sediment component content is not the same as the change trend of the adsorption amount of Cu and Zn, and the compound effect between the components affects the adsorption amount. The adsorption of Cu by the four groups of sediments after different treatments is more in line with the Freundlich isotherm adsorption model; When adsorbing Zn, the untreated and removed organic matter and iron-aluminum oxide group are in good agreement with the Freundlich model, while the organic matter-removed group and the iron-aluminum oxide removal group are more consistent with the Langmuir isotherm adsorption model; The adsorption contribution rate of organic–inorganic composites in sediments is not a simple addition of organic matter and iron-aluminum oxides, but a more complex quantitative relationship.

## Introduction

Energy flow and material circulation in estuaries, the transitional areas between lakes and rivers, are strong. Estuarine circulation, which results in river sediment redistribution, water salinity, redox processes, and pH, affects the mobility (including dissolution, deposition, and diffusion) and spatial distribution of metals^1^. The amount of heavy metals entering Dianchi Lake varies greatly with the seasons due to limited surface runoff during the arid dry season and considerably higher surface runoff in the heavy rains of the rainy season. In addition, as the urbanization of the rivers flowing into Dianchi Lake continues to increase year by year, pollution sources are also increasing. The large amounts of heavy metals carried into Dianchi Lake readily accumulate in the estuary^2^. Previous studies on heavy metals in Dianchi Lake focusing on the spatial distribution, morphological analysis and ecological risk assessment of heavy metals^3^ are mostly based on the direction of adsorption of heavy metals on the sediments. Adsorption is the dominant process of pollutant transfer to sediments^4,5^, directly affecting the migration, transformation, bioavailability, solubility, and activity of heavy metals in the environment, as well as the concentration and bioavailability of heavy metal ions in sediments^6^. Therefore, exploring the adsorption mechanism and distribution characteristics of heavy metals in estuarine sediments is conducive to formulating corresponding prevention and control measures according to local conditions, as well as to improving the understanding of the filtering and purification of heavy metal pollutants in wetland ecosystems and their role in secondary pollution^7^.

Natural water sediments are mainly composed of minerals, with clay minerals (illite, kaolinite, and montmorillonite) as the core substances. Metal oxides (iron oxide, alumina, and manganese oxide) and organic matter (OM, mainly humic acid and tannin acid) bind to the surface of core mineral particles and form flocculent aggregates, which play an indispensable role in the adsorption process of metal pollution^8,9^. Therefore, relevant scholars have performed much research on the role of sediment components in the process of adsorbing heavy metals. Typically, the role of single components in adsorption is studied by artificially synthesizing single sediment components^10,11^ or by selectively removing certain components (e.g., OM or iron oxide) from the sediment^11,12^. However, the study of single components overlooks the influence of organic–inorganic composites in multi-component sediment^13^. It is necessary to consider the role of each potentially influential component to comprehensively characterize sediment adsorption. In this study, we endeavoured to further explore the adsorption mechanism of multi-component interactions and improve the understanding of the multi-component complex adsorption system theory.

This study will investigate the spatial distribution characteristics of Cu(II) and Zn(II) adsorption on sediments at the vertical depth of the estuary and the river flow, and the organic matter, metal oxides, organic–inorganic composites of the sediments affect the adsorption of Cu(II) and Zn(II) on the sediments, especially the contribution rate of heavy metals adsorption between organic matter, metal oxides, and organic–inorganic composites relation, in order to understand the sedimentation laws of heavy metals in natural waters in depth, it is helpful to reveal the adsorption mechanism of wetland sediments to heavy metals, and obtain a combination of adsorption influencing factors with stronger adsorption capacity, which is the preparation of the key bottom matrix material in the construction and restoration of wetlands. Provide theoretical basis, especially according to the characteristics of heavy metals adsorbed in the sediments at the mouth of the lake, remediation measures are taken according to local conditions to form a project implementation technical plan for the prevention of heavy metals in natural water.

## Materials and methods

### Sampling area

Dianchi Lake is the largest plateau lake in Yunnan and one of the urban lakes with serious water environmental pollution. The rivers entering the lake have the characteristics of small flow, short flow, high pollution load, poor water quality, and relatively insufficient river self-purification capacity. The rivers with high pollution load in the basin have seriously threatened the ecological security of Dianchi Lake^14^. Among them, Xinhe is a river entering the lake in the south of Dianchi Lake. It flows through urban and rural residential land and commercial land. It is greatly affected by human activities and has serious sediment pollution with a large amount of mud^15^. Therefore, this study chose Xinhe as the entrance to Dianchi The study area of the adsorption capacity of lake and river sediments to Cu and Zn.

### Sediment sampling

**Figure 1:**
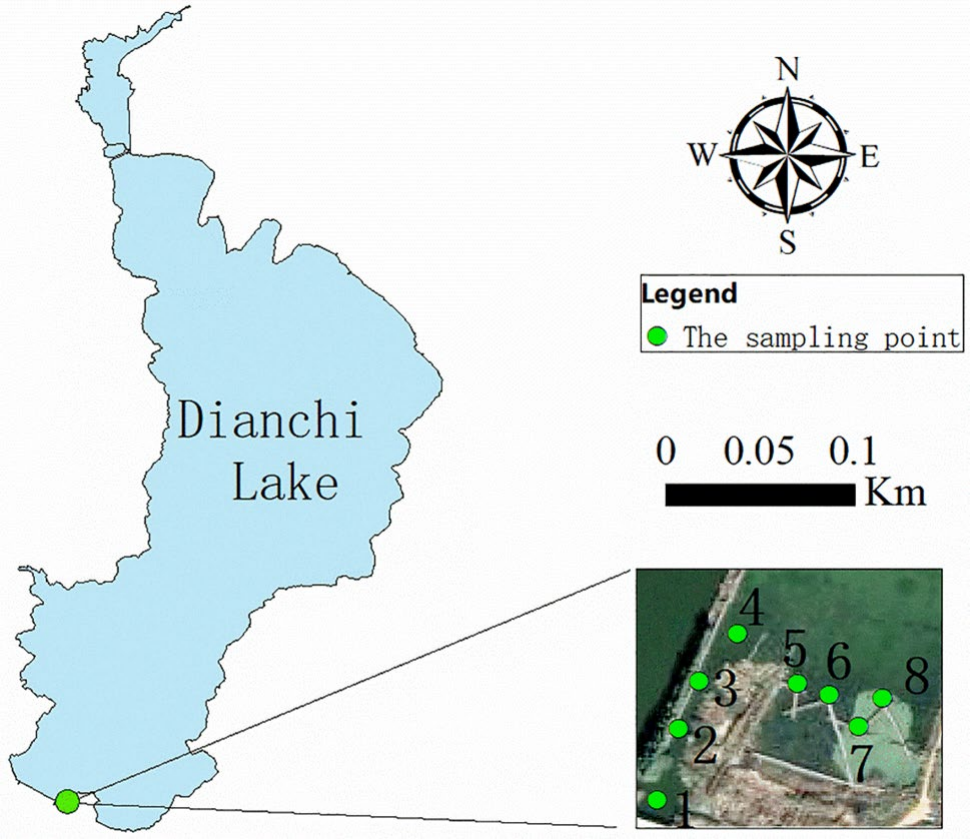
Distribution of sampling points.

The sampling points (Fig. 1) are located in the Xin River next to the Positioning Observation Station of the National Plateau Wetland Research Center. Each sampling point is in the recirculation section, the flow rate is slow, and the water depth is greater than 0.5 m. According to Schiller’s^16^ research, the water flow rate in the recirculation section is slow, organic matter is easy to deposit here, and Cu is fixed in the sediment. Therefore, the sediments in this section can better reflect the influence of human activities on the adsorption capacity of sediments. Sediment samples were collected using a fixed-depth peat drill at 10-cm intervals along the direction of water flow. Three depths were sampled at each interval: (1) 0–10 cm, (2) 10–20 cm, and (3) 20–30 cm. After removal of impurities, air-drying and grinding, samples were passed through a 0.2-mm sieve and stored in sealed Ziplock bags.

The pH of the sediment was measured by the glass electrode method with a water-soil ratio of 1:2.5. Sediment OM was obtained by using a total organic carbon (TOC) analyser to obtain the TOC content and then converting this value with a coefficient. The cation exchange capacity (CEC) was measured by hexaammine cobalt trichloride extraction spectrophotometry. The contents of iron and aluminium oxide were determined by extraction with oxalate-ammonium oxalate solution. Table 1 shows the main physicochemical properties of the studied sediments.

**Table 1:** Basic physical and chemical properties of soil.

pH	OM (g/kg)	CEC (cmol/kg)	Iron oxide (mg/kg)	Aluminium oxide (mg/kg)
8.3	24.23	8.27	107.58	26.99

### Soil sample treatment

Four groups of samples were prepared: (1) untreated, Group A, (2) OM removed, Group B, (3) iron and aluminium oxide removed, Group C, and (4) OM and iron and aluminium oxide removed, Group D.

To prepare Group B, 30% hydrogen peroxide was added to the samples, and the mixtures were placed in a water bath at 90 °C. To prepare Group C, a solution of 16 g of ammonium oxalate and 10.88 g of oxalate was added to 5 g of soil in a 50-ml centrifuge tube. The mixture was stirred in a constant-temperature oscillator at a speed of 300 RPM/min for 48 h. The solution was changed every 24 h, and the soil samples were cleaned with 0.01 mol/L sodium chloride (NaCl) to remove the iron and aluminium oxides in the soil samples. To prepare Group D, both methods were applied.

### Adsorption experiment

A series of experiments was performed on each of the four groups using a range of Cu and Zn concentrations: 3 mg/L, 5 mg/L, 10 mg/L, 20 mg/L, 40 mg/L, and 60 mg/L and 25 mg/L, 50 mg/L, 80 mg/L, 100 mg/L, 160 mg/L, and 200 mg/L, respectively. For each experimental condition, 0.5 g of soil sample was placed in a 50-ml plastic centrifuge tube, 15 ml of sodium nitrate (0.01 mol/L NaNO_3_) was added as the background electrolyte, 1 ml of potassium chloride (2 mol/L KCl) was added to maintain ionic strength, the target concentrations of Cu and Zn were added, the pH was adjusted to a range of 4–8 using nitric acid and sodium hydroxide, and the tubes were placed in a constant-temperature oscillator at a speed of 300 r/min. Then, the tubes were centrifuged at 4000 rpm for 5 min, and the concentrations of Cu and Zn in the supernatant and in the sediment were measured by inductively coupled plasma-optical emission spectroscopy (ICP-OES). The original Cu and Zn contents in the sediment plus the added Cu and Zn minus the contents of Cu and Zn in the supernatant yielded the adsorption capacity.

### Data analysis and statistical treatment

The Freundlich (1) and Langmuir (2) isothermal adsorption models were used to fit the experimental data:

1 $$ {\text{Qe}} = {\text{KfCe}}^{{1/{\text{n}}}} $$

where Qe is the equilibrium adsorption concentration (mg/g), Ce is the equilibrium concentration (mg/L), Kf is the equilibrium adsorption coefficient, and 1/n is the linearity degree of the isothermal adsorption curve:

2 $$ {\text{Qe}} = \frac{{{\text{Q}}\max {\text{KlCe}}}}{{1 + {\text{KlCe}}}} $$

where Qe is the equilibrium adsorption concentration (mg/g), Qmax is the maximum adsorption capacity (mg/g), Kl is the equilibrium adsorption coefficient, and Ce is the equilibrium concentration (mg/L).

Excel 2013 and SPSS 20.0 were used to collate and perform the correlation analysis of the experimental data, Origin 2018 software was used for chart analysis and isothermal adsorption model fitting, and Canoco 5 was used for redundancy analysis.

## Results

### Adsorption capacity and distribution characteristics of Cu and Zn in estuarine sediments

**Figure 2:**
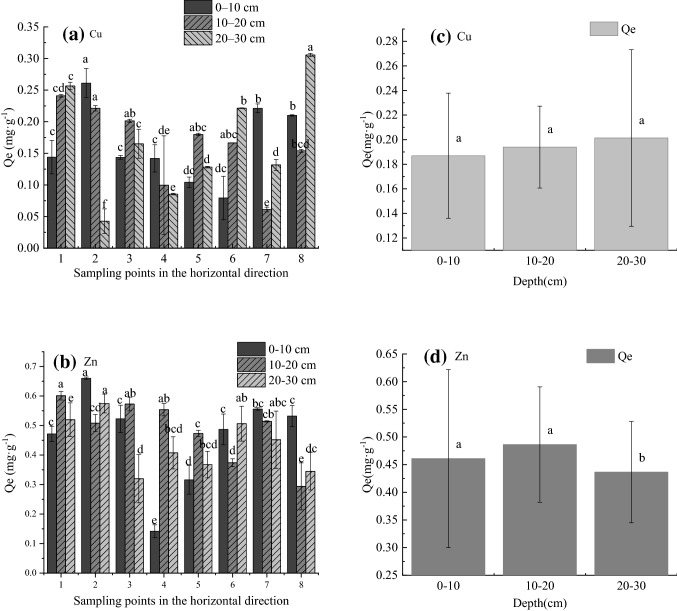
Effects of location along the direction of flow and different depths on Cu(II) and zinc(II) adsorbance changes. (**a**) Adsorption capacity for Cu in the flow direction, N = 8; (**b**) Adsorption capacity for Zn in the flow direction, N = 8; (**c**) Vertical change in the Cu adsorption capacity of sediments, N = 3; (**d**) Vertical change in the Zn adsorption capacity of sediments, N = 3. Different lowercase letters represent difference by Tukey test at 5% probability.

Along the flow of the river to the estuary, the change trend of Cu(II) and Zn(II) adsorption of sediments at the depth of 0–10 cm shows that the positions 1, 2, 6, 7, and 8 are higher than other positions. At 10–20 cm, both Cu and Zn absorption decreased. At 20–30 cm, Cu adsorption gradually increased, while Zn adsorption gradually decreased with distance from the river (Fig. 2). In general, there is a big difference in the adsorption of Cu(II) and Zn(II) by the sediments in the direction of the water flow. According to Tukey's test (Table 2), the changes in the adsorption of Cu(II) and Zn(II) by the sediments have shown significant changes (*P* < 0.05).

**Table 2:** Significance of the difference in the amount of Cu and Zn adsorbed by sediments in the direction of water flow (between groups).

	Cu	Zn
0–10 cm	0.00	0.00
10–20 cm	0.00	0.00
20–30 cm	0.00	0.00

From Fig. 2c,d and Table 3 on the depth, there is no statistical difference in the amount of Cu(II) adsorbed by the three groups of sediments at different depths (*P* > 0.05), and the change trend is not significant. The Zn(II) adsorption amount changes significantly, except that there is no significant difference between 0–10 cm and 10–20 cm (*P* = 0.73), and the other depths are all *P* < 0.05, which is a big difference, and the change trend is 0–10 cm ≈10–20 cm > 20–30 cm, showing a higher adsorption capacity of surface and middle sediments, while a lower adsorption capacity of middle sediments.

**Table 3:** Significant difference in the adsorption of Cu and Zn by sediments at different depths.

	Cu			Zn		
	0–10 cm	10–20 cm	20–30 cm	0–10 cm	10–20 cm	20–30 cm
0–10 cm	/	0.732	0.83		0.73	0.046
10–20 cm	/	/	0.983	/	/	0.006
20–30 cm	/	/	/	/	/	/

### Main characteristics of estuary sediments

The content of each component of the sediment was linearly fitted with the distance from the river (Table 4), and the slope of the fitted line was used to characterize the change trend of each component, with a positive slope indicating an increase in concentration and a negative slope indicating a decrease. At 0–10 cm, OM and CEC increased with distance from the river, while iron and aluminium oxides decreased. At 10–20 cm and 20–30 cm, all components, except for CEC, decreased with increasing distance from the river.

**Table 4:** Slopes of the linear fitting of the content of each sediment component in the direction of deep-water flow.

Depth	OM	CEC	Iron oxide	Aluminium oxide
0–10 cm	25.2	0.4	− 4.83	− 2.2
10–20 cm	− 2.72	0.42	− 14.61	− 0.87
20–30 cm	− 1.74	0.77	− 19.67	− 1.2

**Figure 3:**
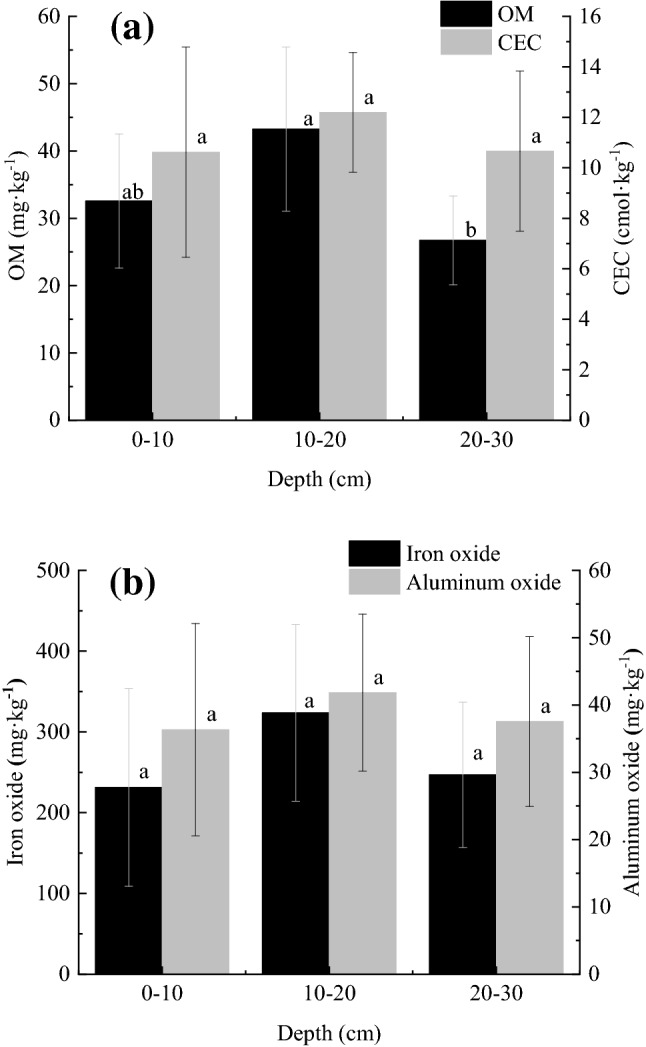
Vertical distribution of each sediment component. (**a**) Vertical distribution of OM content and CEC at three depths, (**b**) Vertical distribution of the adsorption capacities for iron and aluminium oxides at three depths. Different lowercase letters represent difference by Tukey test at 5% probability, N = 3.

Each component in the sediment, except for OM, which has a large difference between the depth of 10–20 cm and 20–30 cm (Fig. 3),The difference of the remaining components at each depth is small (*P* < 0.05), which is similar to the distribution of the adsorption amount of Zn.

### Statistical analysis of isothermal adsorption

**Figure 4:**
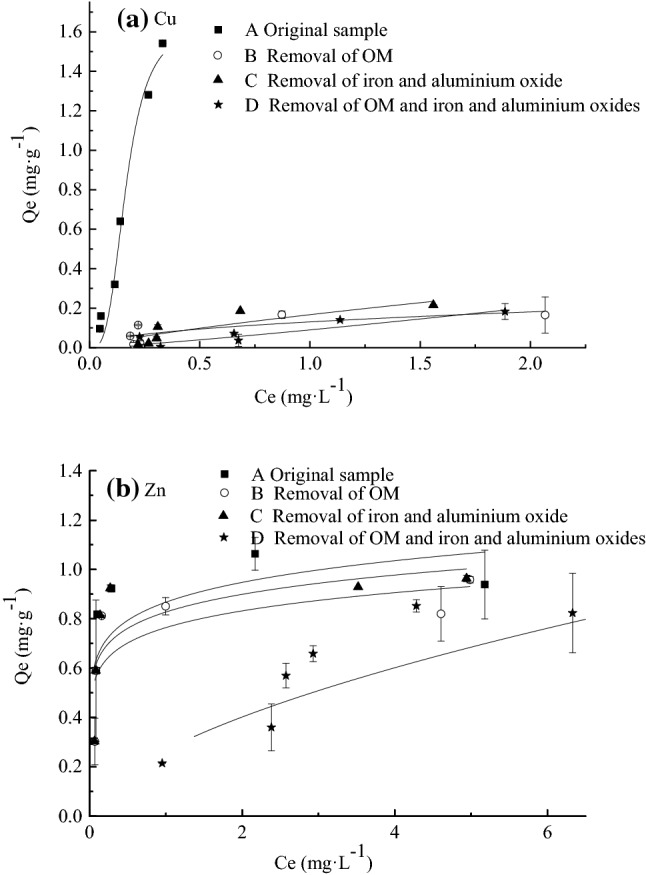
Isotherm adsorption curves. (**a**) Cu, (**b**) Zn.

As shown in Fig. 4 and Table 5, the difference in the adsorption of Cu in group A was much greater than that in the other three groups. The adsorption of Cu in group A increased to 1.54 mg/g, while the highest adsorption capacities of groups B, C, and D were 0.17, 0.22, and 0.18 mg/g, respectively. The isotherm adsorption curve gradually stabilized, and the adsorption process tended to reach a balance. Adsorption equilibrium was not reached for Group A, the untreated group.

**Table 5:** Cu(II) isotherm adsorption model parameters for four groups of sediments from the mouth of Dianhu Lake.

	Freundlich			Langmuir		
	K_f_	1/n	R^2^	K_l_	Qmax	R^2^
A	6.72	1.29	0.99944	400.30	1.63	0.97581
B	0.13	0.47	0.99721	/	/	/
C	0.17	0.76	0.99463	0.53	0.51	0.99539
D	0.1	1.02	0.99876	/	/	/

The correlation coefficients (R^2^) indicate that all four groups showed a good fit with the Freundlich isothermal adsorption model, indicating that the adsorption of Cu was dominated by multi-layer heterogeneous adsorption. The adsorption capacity of the four samples followed the order A > C > B > D, and the order of the K value (representing the adsorption force in the Freundlich model) was also A (6.72) > C (0.17) > B (0.13) > D (0.1).

The fact that 1/n > 1 (1.02) in the isotherm adsorption curve of Group D indicates that Group D follows a linear adsorption process and that the adsorption process is difficult to carry out. Additionally, given that 1/n > 1 (1.29) for Group A, it is possible that Group A is far from reaching adsorption equilibrium, making the adsorption isotherm curve present a similar linear adsorption process. For the other two groups, 1/n < 1 (0.47 and 0.76), indicating that the adsorption process is nonlinear adsorption.

As shown in Fig. 4 and Table 6, the isothermal adsorption curves of Groups A, B, C and D gradually stabilized for Zn, and the adsorption process tended to reach equilibrium. The Freundlich model provided consistent results for Group A and Group D, indicating that their adsorption process was dominated by multilayer heterogeneous-phase adsorption, while Group B and Group C were more in line with the Langmuir isothermal adsorption model. The four groups of samples presented values of 1/n < 1 (0.04, 0.03, 0.03, 0.63) in the Freundlich model, indicating that the adsorption process was non-linear. Nevertheless, the adsorption results showed similar trends for Zn and Cu, with the K value following the order Groups A > C > B > D.

**Table 6:** Zn(II) isotherm adsorption model parameters for four groups of sediments from the mouth of Dianhu Lake.

	Freundlich			Langmuir		
	K_f_	1/n	R^2^	K_l_	Qmax	R^2^
A	0.94	0.04	0.9934	/	/	/
B	0.85	0.03	0.99915	15.98	0.92	0.69137
C	0.91	0.03	0.99472	15.47	1.00	0.72573
D	0.29	0.63	0.99807	/	/	/

## Discussion

Correlation analysis between the content of each component and the adsorption capacity of the sediment in the direction of water flow was carried out. The correlation between the adsorption amount and the OM content, CEC, and iron and aluminium oxide contents did not correspond to the change trend of the adsorption amount. It can be seen from Table 7 that at 0–10 cm, the adsorption of Cu in the sediment had a high correlation with aluminium oxide (0.907), but the change trend of the adsorption amount was not significant. At 0–10 cm, the correlation of adsorption capacity with CEC was 0.943, but the adsorption capacity did not increase with increasing CEC. At 10–20 cm, the adsorption of Zn was correlated with OM (0.604), but it did not increase with increasing OM.

**Table 7:** Correlation between adsorption capacity and sediment components at three depths along the direction of water flow.

Depth	Cu				Zn			
	OM	CEC	Iron oxide	Aluminium oxide	OM	CEC	Iron oxide	Aluminium oxide
0–10 cm	0.626	0.619	0.721	0.907	0.799	0.943	0.499	0.518
10–20 cm	0.044	− 0.297	− 0.2	0.037	0.604	− 0.444	0.411	0.217
20–30 cm	− 0.699	0.361	0.177	0.147	0.115	− 0.108	0.479	0.489

These results showed that a high content of a certain sediment component does not mean that this component is the only factor driving the adsorption process. The overall effect may be due to the change in the surface potential of sediment particles. Studies have shown that (^17^ after removing iron and aluminium oxides in soil, the amount of soil adsorption of Pb^2+^ and Cd^2+^ increases. This increase occurs because Pb^2+^ and Cd^2+^ can interact with the remaining citrate in solution to form acid salt complexes. The complexes are adsorbed on the surface of the soil particles, increasing the negative potential. Another factor may be that even if the content of a certain component is high, other intertwined components may block or overlap adsorption point sites. A study found^18^ that iron oxide can mask some charge sites of soil particles.

To further explore the relationship between OM content, CEC, iron and aluminium oxide contents and adsorption capacity in the direction of water flow, redundancy analysis (RDA) was applied using Cu and Zn as species and the OM content, CEC, and iron-aluminium oxide content in the sediment as environmental factors. The analysis results showed that the correlation between the adsorption of Cu and Zn and each component of the sediment was different.

**Figure 5:**
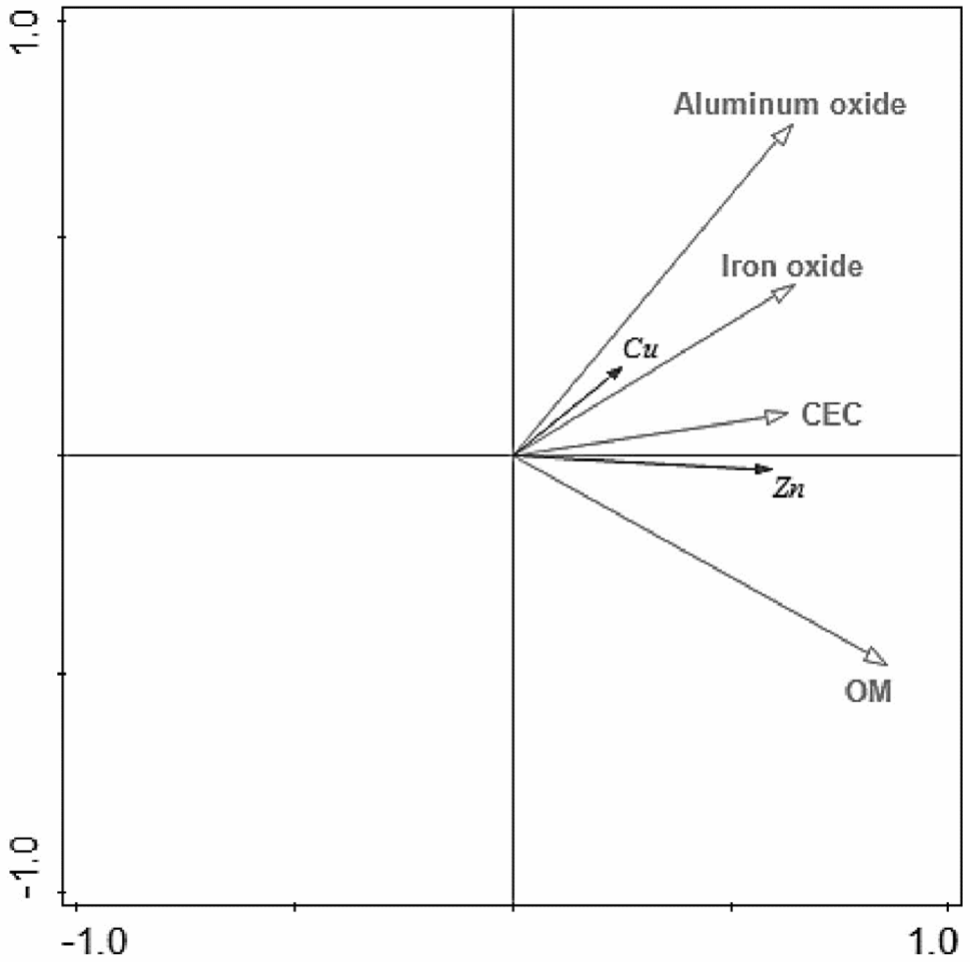
Redundancy analysis diagram of sediment components and adsorption capacity.

Figure 5 shows Cu, iron oxide and aluminium oxide at angles less than 90°, indicating that iron oxide and aluminium oxide were correlated more strongly with Cu adsorption than were other components of the sediment, while OM and iron oxide may be more strongly correlated with Zn adsorption.

Tables 8 and 9 show that the contribution rates of OM and aluminium oxide in the adsorption process were higher than those of CEC and iron oxide. The contribution rate of OM in the entire adsorption process reached 72%, with a maximum significant difference of 0.002. Based on the distribution characteristics of the adsorption amount with increasing distance from the river and the redundancy analysis results for the sediment components and adsorption amounts, the adsorption of Cu in the Dianchi Lake Estuary is strongly affected by iron and aluminium oxides, while the adsorption of Zn is more affected by OM and CEC.

**Table 8:** Redundancy analysis parameters for sediment components involved in adsorption.

	Interpretation %	Contribution rate %	pseudo-F	*P*
OM	20.9	72.0	18.5	0.002
CEC	< 0.1	0.3	< 0.1	0.928
Aluminium oxide	7.7	26.6	7.5	0.008
Iron oxide	0.3	1.1	0.3	0.738

**Table 9:** Correlation between sediment components and adsorption capacity.

		OM	CEC	Aluminium oxide	Iron oxide
Cu	Correlation	0.126	0.173	0.318	0.243
	Significance	0.222	0.092	0.007	0.04
Zn	Correlation	0.441	0.309	0.357	0.372
	Significance	0.000	0.002	0.002	0.001

Different sediment components adsorb different amounts of Cu and Zn. It has been reported^19^ that OM and iron and manganese oxides have a relatively high level of adsorption of Cu. Feng Jun et al.^20^ also found that iron oxide strongly adsorbs Zn.

In summary, the components of the sediment are entangled with each other, shielding or overlapping adsorption sites, so the adsorption amount at each depth is not consistent with the OM content, CEC, iron and aluminium oxide contents or the change trend of the adsorption amount and the adsorption capacity does not increase with increases in OM content, CEC, or Fe-Al oxide content. The sediment components exhibit differential adsorption of heavy metals: iron-aluminium oxide contributes more to the adsorption of Cu(II), and OM and iron oxide contribute more to the adsorption of Zn(II).

Different components of sediment have different adsorption properties for heavy metals; iron and aluminium oxides contribute more than other components to Cu adsorption, while OM and iron oxide contribute more to Zn adsorption.

Previous research results showed that there is a certain linear positive correlation between the OM content, CEC, and metal oxide contents in sediments and the adsorption amount^21–23^. The results are shown in Table 10. The R^2^ between each component and the amount of adsorption is not high, indicating that the amount of adsorption does not depend on a single component, but is the result of the combined effect of multiple components.

**Table 10:** Regression equations between sediment components and adsorption capacity.

	Cu	Zn
OM	Y = − 5.064E−006x^3^ − 0.001x^2^ − 0.043x − 0.696 R^2^ = 0.031	Y = 6.259E−007x^3^ + 0.064x − 1.422 R^2^ = 0.181
CEC	Y = − 0.014x^2^ + 0.278x − 1.165 R^2^ = 0.042	Y = 0.002x^3^ − 0.079x^2^ + 0.979x − 3.243 R^2^ = 0.277
Fe	Y = − 1.371E−007x^3^ + 8.939E−005x^2^ − 0.013x + 0.031 R^2^ = 0.110	Y = − 7.179E−008x^3^ + 5.867E−005x^2^ − 0.009x − 0.08 R^2^ = 0.188
Al	Y = − 0.001x^2^ − 0. 099–1.517 R^2^ = 0.098	Y = − 6.344E−005x^3^ − 0.06x^2^ − 0.159x + 0.886 R^2^ = 0.128

In the adsorption of Cu and Zn, as the equilibrium adsorption concentration increases, the competition for adsorbate molecules to occupy the adsorbent sites becomes more intense. When a high-binding-energy adsorption site is close to its full energy, non-specific adsorption increases, and the adsorption rate gradually slows^24^. The iron-aluminium oxides and OM in group B, group C, and group D masked adsorption sites; thus, the K values were much lower in these groups than in group A (400.3). As the adsorption equilibrium concentration increased, the number of adsorption sites of groups B, C, and D decreased faster than those of group A, and the growth rate of the adsorption capacity slowed faster than that of group A. Relevant studies have shown^25^ that as the equilibrium concentration increases, the adsorption capacity increases, and the adsorption curve shows a sharp increasing trend at low concentrations. However, the adsorption potential is limited, and when the concentration of heavy metal ions is high, the electrokinetic potential and electrical properties of colloidal particles are reduced, which reduces the stability of heavy metal-colloid-soil aggregates, and the curve gradually flattens^26^. The decrease in adsorption capacity with the increase in adsorption equilibrium concentration may be caused by the decrease in adsorption sites and the limited adsorption capacity.

In practice, the surface of sediment particles is uneven, which makes the number and distribution of adsorption sites uneven. The adsorption isotherms of the sediments for Cu and Zn were more consistent with the Freundlich adsorption isotherm model than the Langmuir model, indicating that the adsorption process follows multi-layer adsorption. The surface of sediment particles is not uniform, so the fit of the Freundlich isotherm adsorption model aligns with reality; Mustapha 's et al.^27^ research results are similar. The order of the adsorption amounts of the samples corresponds to the order of their K values (because some samples could not be fitted by the Langmuir isotherm adsorption model, the Freundlich adsorption isotherm model K value was used for comparison), which is similar to most research results^28^. The K value was used as an index to measure the strength of the adsorption capacity: the larger the value was, the greater the adsorption force of the sediment for Cu and Zn. Among groups B, C, and D, the K values of group C (0.17 and 0.83) were the largest, and the K values (0.09 and 0.27) of group D were the smallest, indicating that iron-aluminium oxides and OM are important influencing factors in the adsorption process and that iron-aluminium oxide has a stronger adsorption capacity than OM for heavy metals.

In the past, studies on changes in adsorption characteristics before and after the removal of soil and sediment components have focused on the changes in the isotherm adsorption equation or kinetic adsorption equation of each single component^29^, but they have not considered combined effects. Individual compound effects and isolated studies of inorganic colloids (minerals) or organic colloids (OM) cannot reflect true sediment systems.

**Figure 6:**
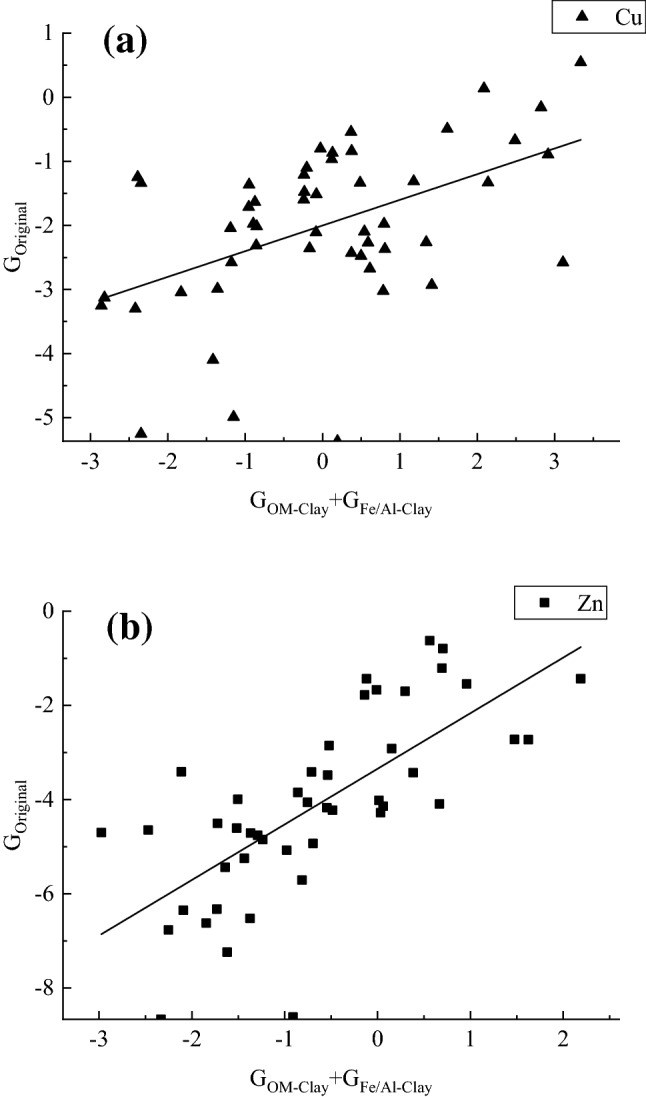
Quantitative relationship between the contribution rates of OM, iron-aluminium oxides, and organic–inorganic composites to the adsorption of Cu(II) and Zn(II). (**a**) Cu, (**b**) Zn.

The contribution rate of each component to the adsorption of Cu and Zn in the sediments was calculated according to Eq. (3):

3 $$ {\text{G}} = \left( {{\text{Q}}_{{{\text{not}}\;{\text{removed}}}} - {\text{Q}}_{{{\text{removal}}}} } \right)/{\text{Q}}_{{{\text{removal}}}} * 100\% $$

where G is the rate for a particular component, (Q _not removed_) is the adsorption without removal of that component and (Q _removal_) is the adsorption with the removal of that component. To obtain the quantitative relationship between the fitting curve and G_OM-IAO_, G_OM_ and G_IAO_, in Fig. 6, the contribution rate of OM (G_OM_) plus the contribution rate of iron and aluminium oxide (G_IAO_) was used as the X-axis, and the contribution rate of OM-iron and aluminium oxide complexes (G_OM-IAO_) was used as the Y-axis. The clear positive linear correlation between G_OM-IAO_, G_OM_ and G_IAO_ indicates that OM-iron-aluminium oxide composites play a role in the adsorption of Cu and Zn. The simple addition of iron and aluminium oxides results in a certain quantitative relationship for the adsorption of Cu (4):

4 $$ {\text{G}}_{{\text{OM - IAO}}} = \left( {{\text{G}}_{{{\text{OM}}}} + {\text{G}}_{{{\text{IAO}}}} } \right) * 0.4 - 2 $$

as well as for the adsorption of Zn (5):

5 $$ {\text{G}}_{{\text{OM - IAO}}} = \left( {{\text{G}}_{{{\text{OM}}}} + {\text{G}}_{{{\text{IAO}}}} } \right) * 1.18 - 3.35 $$

The organic/inorganic composite content was not equal to the sum of OM and metal oxides, presumably because hydrogen bonding, ion exchange and hydrophobic forces, such as anion adsorption mechanisms, embedded the composites in the mineral surface and between layers of swollen clay mineral crystals^30,31^, affecting the mineral cementation degree and thus colloid stability^32^. In addition to directly participating in the formation of complexes, the strong surface activity of iron and aluminium oxide can form bridges with OM and stabilize colloids through coordination exchange or the formation of ionic bonds^33^. In this way, these components combine to form organic and inorganic complexes that form the core and structure of the soil^34–36^.

**Figure 7:**
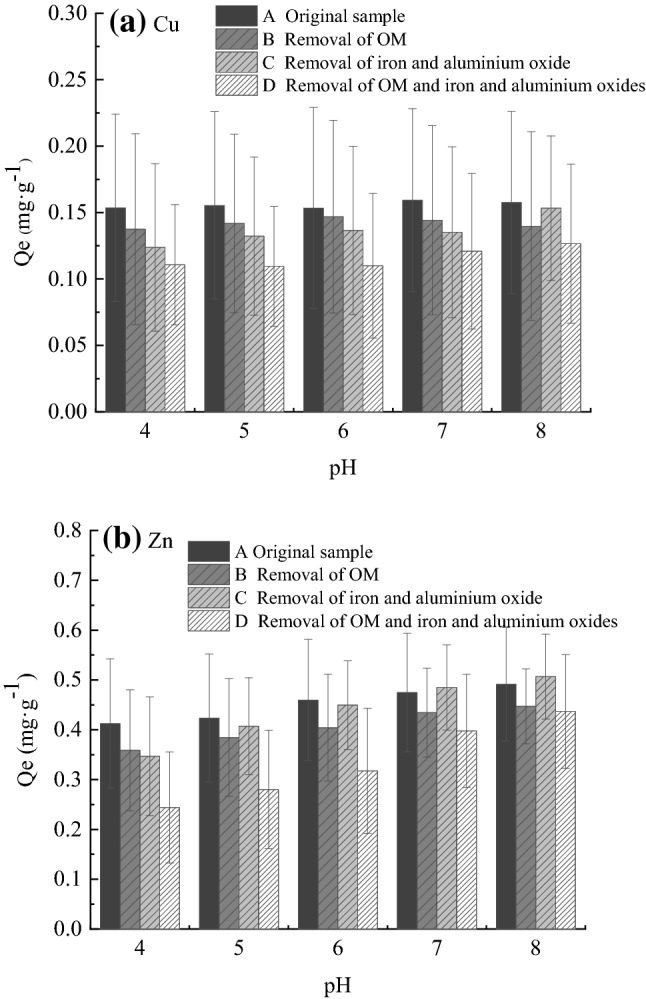
Relationship between adsorption capacity and pH. (**a**) Adsorption capacity for Cu of four groups of samples at different pH values; (**b**) Adsorption of Zn to four groups of samples at different pH values.

To further compare the adsorption capacity differences between OM-iron-aluminium oxide composites, OM and iron-aluminium oxide, we compared the four groups of sediment samples under different pH values and adsorption quantity changes (Fig. 7). We found that at different pH values, the sediment Cu and Zn adsorption performance for group A was greater than that of the other three groups. Similar results were reported by Perez-Novo et al^37^.

In group A, OM and iron and aluminium oxides formed organic–inorganic complexes, increasing the sediment surface area and surface activity and enhancing the adsorption capacity^38^. In addition, Mg(II) and Fe(II) compounds in sediments may be dissolved due to the presence of a large amount of H^+^ in low-pH environments, thus competing for adsorption sites, or Cu could form hydroxylates with increasing pH^24,39,40^. In group D, OM and iron and aluminium oxide were removed simultaneously, which greatly reduced the number of active sites on the sediment surface and exposed the silicic skeleton of the sediments, making adsorption more susceptible to the influence of pH value.

The surface properties of iron oxides change when OM and iron and aluminium oxides form organic–inorganic complexes. First, the decrease in zeta potential indicates that the negative charge on the surface increases, which is conducive to improving the adsorption of cations. Zhou et al.^41^ found that a large amount of dissociated humic acid may cover the surface of iron oxide, reducing its surface electric potential and making the zeta potential drop. Second, OM and iron aluminium oxide have a large number of adsorption sites^42^. OM adsorbs heavy metals by means of ion exchange, surface complexation and precipitation with carboxyl, hydroxyl and other functional groups in OM with a large number of negative charges, while humic acid and fulvic acid in OM adsorb heavy metals through complexation^11,43^. Metal oxides, represented by iron and aluminium oxides, have variable charges, which can be replaced by H^+^ ions through the surface -OH groups and adsorbed on negatively charged sites^44^ or react with surface groups to form complexes^45^.

In summary, the organic–inorganic composites in the sediments did not correspond to the simple addition of OM and iron-aluminium oxides. Their contribution rates to the adsorption of Cu and Zn were G_OM-IAO_ = (G_OM_ + G_IAO_) * 0.4–2 and G_OM-IAO_ = (G_OM_ + G_IAO_) * 1.18–3.35. When organic–inorganic complexes are formed, the zeta potential decreases, the surface negative charge increases, and the number of adsorption sites such as functional groups and variable charges increase, making the adsorption capacity of organic–inorganic complexes for Cu and Zn significantly higher than those of OM and iron and aluminium oxides.

## Conclusion

Along the direction of water flow, with increasing distance from the river, the adsorption of Cu and Zn showed no significant change trend at a depth of 0–10 cm, a decreasing trend at 10–20 cm, and an increasing trend at 20–30 cm. In terms of depth, the adsorption capacity presented a trend of 0–10 cm ≈10–20 cm > 20–30 cm.

1. The components of the sediment have different adsorption differences for heavy metals, so that the contribution rate of iron-aluminum oxide in the adsorption of Cu(II) in the sediment is greater than that of organic matter and CEC. Organic matter and iron oxide contribute more to the adsorption of Zn(II) than CEC and aluminum oxide. At the same time, the adsorption does not depend on a single component, and the adsorption is the result of the combined effect of multiple components.
2. Among the samples in group A (without any treatment), Group B (removal of OM), Group C (removal of iron and aluminium oxide), and Group D (removal of both OM and iron and aluminium oxide), the Freundlich isothermal adsorption model provided the best fit for the adsorption of Cu. In the adsorption of Zn, group A and Group D were in good agreement with the Freundlich model, while group B and Group C were in better agreement with the Langmuir isothermal adsorption model.
3. The organic–inorganic composites in the sediments do not simply correspond to the sum of OM and iron-aluminium oxides. Their contribution rates in to adsorption of Cu and Zn are G_OM-IAO_ = (G_OM_ + G_IAO_) * 0.4–2 and G_OM-IAO_ = (G_OM_ + G_IAO_) * 1.18–3.35, respectively. The adsorption capacity of organic–inorganic composites is significantly higher than that of OM and iron aluminium oxide.
